# Knowledge, attitude, and intention to accept COVID-19 vaccine among patients with chronic diseases in southern Ethiopia: Multi-center study

**DOI:** 10.3389/fpubh.2022.917925

**Published:** 2022-09-29

**Authors:** Getachew Asmare Adella, Kelemu Abebe, Natnael Atnafu, Gedion Asnake Azeze, Tamiru Alene, Simegn Molla, Gizachew Ambaw, Tekalign Amera, Amanuel Yosef, Kirubel Eshetu, Adisu Yeshambel, Dabere Nigatu, Endeshaw Chekol Abebe, Belete Birhan, Yibeltal Assefa

**Affiliations:** ^1^School of Public Health, College of Health Science and Medicine, Wolaita Sodo University, Sodo, Ethiopia; ^2^School of Midwifery, College of Health Science and Medicine, Wolaita Sodo University, Sodo, Ethiopia; ^3^Department of Nursing, College of Health Science, Injibara University, Injibara, Ethiopia; ^4^Department of Adult Health Nursing, College of Health Science, Debre Tabor University, Debre Tabor, Ethiopia; ^5^School of Nursing, College of Health Science and Medicine, Wolaita Sodo University, Sodo, Ethiopia; ^6^School of Public Health, College of Health Science and Medicine, Bahir Dar University, Bahir Dar, Ethiopia; ^7^Department of Medical Biochemistry, College of Health Sciences, Debre Tabor University, Debre Tabor, Ethiopia

**Keywords:** COVID-19 vaccine, knowledge, attitude, intention, chronic disease

## Abstract

**Background:**

Most of the COVID-19 fatal cases and severe illnesses like acute respiratory distress syndrome occur in older adults and other people who have underlying medical comorbidities. Understanding patients with chronic disease' knowledge, attitudes, and intention to take the COVID-19 vaccine and related factors are necessary to control the mortality of COVID-19 infection. Therefore, this study aimed to assess knowledge, attitudes, and intention to take the COVID-19 vaccine among patients with chronic disease in Southern Ethiopia.

**Methods and materials:**

A facility-based cross-sectional study was conducted among 409 patients with chronic diseases having a follow-up at the hospitals of the Southern region of Ethiopia from November 14, 2021, to December 24, 2021. A structured, interviewer-administered questionnaire was used to collect data. Bivariate and multivariable logistic regression was conducted to show the association of variables with knowledge, attitude, and intention to take the COVID-19 vaccine. The associations of variables were declared with the use of a 95% CI and *P*-value < 0.05.

**Results:**

Overall, 79.2, 70.9, and 58.2% of participants had good knowledge, favorable attitude, and intent to take the COVID-19 vaccine, respectively. Age ≥49 years old (AOR = 1.643; 95% CI = 1.008–3.060) and college and above level of education (AOR = 3.002; 95% CI = 1.897–5.021) were found to be significantly associated with knowledge about COVID-19 vaccine. College and above level of education (AOR = 1.510; 95% CI = 1.002–3.975) and good knowledge (AOR = 3.560; 95% CI = 1.481–6.120) were found to be significantly associated with intention to take COVID-19 vaccine.

**Conclusion and recommendation:**

Intention to take the COVID-19 vaccine was low among patients with chronic diseases to achieve herd immunity. Therefore, a holistic and multi-sectoral partnership is necessary for a successful COVID-19 vaccination campaign. Further health education and communication are very crucial methods to improve vaccine acceptance and lastly to achieve herd immunity.

## Introduction

Most of the fatal cases and severe illnesses like acute respiratory distress syndrome (ARDS) occur in older adults and other people who have underlying medical comorbidities like diabetes, cancer, hypertension, heart, lung, and kidney diseases (1–3). The impacts of COVID-19 on patients with chronic disease are not only restricted to direct effects but also in an indirect manner. Resources at all levels have been shifted away from patients with chronic disease management and prevention to pandemic management particularly, in low and middle-income countries, including Ethiopia (4).

African healthcare systems are not well-equipped to tackle this pandemic (5). Since African nations are more vulnerable to disease spread due to limited health infrastructure and training, their inability to obtain the vaccine easily increased the risk of disease spread. However, many developed countries ordered most of the vaccine supplies, but vaccine-related costs and transfer issues can also further delay vaccination procedures for African people as far as late 2021 or early 2022 (6).

At least seven separate vaccines across three channels are administered in countries as of February 18, 2021. Vaccination is prioritized for vulnerable groups in all countries. Simultaneously, more than 200 additional vaccine candidates are being developed, with more than 60 of them in clinical trials (7). Ethiopia received 2.184 million doses of the COVID-19 vaccine on March 7, 2021 (8). Public media sources recently revealed that about 1.9 million people in Ethiopia received their first dose of a COVID-19 vaccine (9).

Based on prior studies, knowledge about the COVID-19 vaccine was in the range of 35.5%−91.15% (10–17), and intention to take the vaccine was within the range of 31.4–64.9% (18–25).

According to studies conducted in various countries around the world, age, occupational status, educational status, income, perceived risk of COVID-19 infection, attitude, knowledge of COVID-19, being sick with COVID-19, and the presence of chronic disease are the most important predictors of intention to use COVID-19 vaccine (10, 26, 27).

There is no study done so far on knowledge, attitude, and intention to take the COVID-19 vaccine among patients with chronic disease in Ethiopia. Therefore, this study aimed to assess knowledge, attitude, and intention to take the COVID-19 vaccine among chronic disease patients in Southwest Ethiopia.

## Methods and materials

### Study design, setting, period, and population

An institutional-based cross-sectional study was conducted from November 14, 2021, to December 24, 2021, in Wolaita zone hospitals, southern Ethiopia. Wolaita zone is located 328 km south of the capital city Addis Ababa. Based on the 2007 census conducted by the Central Statistical Agency (CSA), the population of the Wolaita zone was projected to be 1,901,112. Wolaita zone is administratively divided into sixteen districts and six town administrations. Currently, there are 9 hospitals and 68 health centers. Those nine hospitals serve as the major referral and reference hospitals in the Woliata zone. The names of the hospitals with their respective total number of patients with chronic diseases attending for follow-up service in 2021 are illustrated as follows: Gesuba Primary Hospital (412), Boditi Primary Hospital (128), Bitena Primary Hospital (316), Halale Primary Hospital (169), Belle Primary Hospital (368), WSUTRH (923), Bombe Primary Hospital (241), Christian Hospital (188), and Dubo Hospital (220). All patients with the chronic disease having a follow-up at the hospitals during the study period were included, whereas patients with chronic diseases who were unable to respond during data collection, <18 years, and health professionals were excluded from this study (Wolaita Zone Health Department Annual Report, 2021).

### Sample size and sampling procedure

The required sample size was calculated by using a single population proportion formula [*n* = Z^2^ *p* (1-p)/d^2^] with an assumption of 95% confidence interval (CI), 5% margin of error, 50% proportion of knowledge, attitude, and intention to take COVID-19 vaccine among patients with chronic diseases (because there is no study done so far). The sample size was found to be 384. Finally, by adding a 10% non-response rate, the final sample size was 422. Based on the number of patients with chronic diseases follow-up in the hospitals, staffing with internist/GP, and availability of diagnostic materials, five hospitals were selected for study (Gesuba Primary Hospital, Bitena Primary Hospital, Belle Primary Hospital, WSUTRH, Bombe Primary Hospital). According to the report obtained from registration records, at the time of the study, about 2,965 patients with chronic diseases different cases were registered for follow-up in the total selected hospitals. Of the 2,965 patients with chronic diseases on follow-up, 590 were scheduled to present for care or pharmacy pick-ups between November 14, 2021 and December 24, 2021, in selected hospitals, and the samples were allocated proportionally to each selected hospital. Participants who fulfilled the inclusion criteria were offered to participate. A systematic random sampling method was conducted to select the study participants.

### Data collection tools, procedures, measurements, and quality control

A structured interviewer-administered questionnaire was used for data collection. The questionnaire was adopted and modified from different works of literature (10, 13, 17, 28–30). Before distribution, its validity was checked by a panel of experts in the field, and ascertained its content validity. A pretest was also done among 5% of the sample size in Dubo hospital. It was then amended according to the comments raised by the experts and the pretest result. Reliability analysis was done and Cronbach's Alpha had been 0.79, indicating good internal consistency in the responses. It was prepared in the English language, then translated to a local language, and translated back to English to maintain its consistency. The questionnaire had four parts. The first part comprised questions regarding personal socio-demographic information, the second part consisted of knowledge-related items, the third part covered attitude-related items and the last part was intention-related variables. Five diploma nurse data collectors and one-degree nurse supervisors were assigned. The training was given to data collectors and supervisors for 2 days on the way of administering and gathering the questionnaire. The completeness of the questionnaire was also checked before data entry.

Comprehensive knowledge of the COVID-19 vaccine was computed by summing up all relevant nine knowledge-related questions. The correct answer for each item was scored “1” and the incorrect or do not know the answer was scored “0.” Accordingly, respondents who scored greater than or equal to the mean value of the sum of items were thought of as having good knowledge, and respondents who answered less than the mean of the sum of items were thought of as having poor knowledge (13, 30, 31).

The question regarding attitude was six (with a minimum score of six and a maximum score of 30). Five points Likert scale, during which a score of 1–5 was given from strongly disagree to strongly agree supported the score of the attitude. Accordingly, respondents who scored greater than or equal to the mean of the sum of attitude-related questions were thought of as having a favorable attitude, and respondents who answered less than the mean value of the sum of attitude-related questions were thought of as having an unfavorable attitude (13, 29–31).

### Data processing and analysis

The collected data were coded and entered into Epi Data version 3.1 and then exported to SPSS version 23.0 for analysis. Descriptive statistics were done by computing summary statistics like frequency, mean, percentages, and standard deviation, then the results were presented in tables and graphs. Binary logistic regression was done to assess the association between the dependent and the independent variables. Multivariable logistic regression was applied to ascertain the independent effect of every variable on the dependent variable. Multi-collinearity among the independent variables was checked using VIF (2.14) and Hosmer and Lemeshow test was done to assess model goodness of fit (0.89). The final results of the association were presented based on the AOR at a 95% CI level and *p* < 0.05.

### Ethical approval and consent from the participant

Ethical approval was obtained from the Institutional Review Board (IRB) of Wolaita Sodo University. A permission letter was obtained from the Woliata Zone Health Department. The consent form was read to the participants and written consent was obtained from each participant before the interview. Participants were informed as they can skip question/s that they don't want to answer partially or fully and to stop at any time if they want to do so. Confidentiality of the individual information was assured by not recording the identifying information.

## Results

### Socio-demographic and clinical characteristics of respondents

A total of 409 participants participated in this study with a response rate of 97%. The mean age of the participants was 52.78 ± (14.69) years old. The majority of the participants 253 (61.9%) were males. The majority of 334 (81.7%) of the participants lived in the urban area. Regarding educational status, 118 (28.9%), and 174 (42.5%) of the participants were in college and above level and secondary educational status, respectively. Concerning the clinical background, 120 (29.3%), and 104 (25.4%) participants had hypertension and Diabetes mellitus, respectively (Table 1).

**Table 1:** Sociodemographic and clinical characteristics of patients with chronic diseases, southern Ethiopia, 2021 (*N* = 409).

**Variables**	**Category**	**Frequency**	**Percent**
Age	18–34	40	9.8%
	34–49	176	43%
	≥49	193	47.2%
Sex	Male	253	61.9%
	Female	156	38.1%
Marital status	Married	200	48.9%
	Single	35	8.6%
	Divorced	77	18.8%
	Widowed	97	32.7%
Residence	Urban	334	81.7%
	Rural	75	18.3%
Educational status	Primary education (grade 1–8)	117	28.6%
	Secondary education (grade 9–12)	174	42.5%
	College and above level of education	118	28.9%
Monthly income in USD	<$40	153	37.4%
	$40–$80	84	20.5%
	≥$80	172	42.1%
Occupation	Merchant	113	27.6%
	Government employee	127	31.1%
	Private employee	58	14.2%
	Farmer	39	9.5%
	Housewife	37	9.0%
	Others	35	8.6%
Type of chronic diseases	Diabetes mellitus	104	25.4%
	Hypertension	120	29.3%
	Heart diseases	39	9.5%
	Lung diseases	52	12.7%
	DM and hypertension	73	17.8%
	Others	21	5.1%

### Sources of information and knowledge about COVID-19 vaccines

Three hundred forty-nine (85.3%) of participants ever heard about the COVID-19 vaccine. The major sources of information were from national TV/radio (64.7%) and health providers (15.2%). Overall, 324 (79.2%) participants had good knowledge about COVID-19 vaccines (Table 2).

**Table 2:** Source of information and knowledge about COVID-19 vaccine among patients with chronic diseases, southern Ethiopia, 2021 (*N* = 409).

**Variables**	**Category**	**Frequency**	**Percent**
Ever heard about COVID 19 vaccine	Yes	349	85.3%
	No	60	14.7%
source of information	From news from national TV/radio	226	64.7%
	From government agencies	23	6.6%
	Social media (Facebook, telegram	29	8.3%
	Discussion amongst friends and families	18	5.2%
	Health care providers	53	15.2%
COVID 19 vaccines use inactivated coronavirus as the antigen^*^	Correct	332	81.2%
	Wrong/do not know	77	18.8%
COVID-19 vaccine can also protect us from influenza	Correct	315	77%
	Wrong/do not know	94	23%
COVID-19 vaccines use genetic material from coronavirus as the active ingredient^*^	Correct	295	72.1%
	Wrong/do not know	114	27.9%
COVID 19 vaccines will be given *via* injection^*^	Correct	337	82.4%
	Wrong/do not know	72	17.6%
Everyone including children can receive COVID-19 vaccination	Correct	289	70.7
	Wrong/do not know	120	29.3%
COVID-19 vaccination may protect other people who do not receive the vaccine^*^	Correct	346	84.6%
	Wrong/do not know	63	15.4%
COVID-19 vaccine protects the receiver from getting COVID-19 infection^*^	Correct	352	86.1%
	Wrong/do not know	57	13.9%
COVID-19 vaccines do not have side effects	Correct	311	76%
	Wrong/do not know	98	24%
COVID-19 vaccine stimulates our body to produce antibodies^*^	Correct	339	82.9%
	Wrong/do not know	70	17.1%
Knowledge about vaccine	Good	324	79.2%
	Poor	85	20.8%

* Yes was the correct answer.

### Factors associated with knowledge about the COVID-19 vaccine among patients with chronic diseases

Age ≥49 and educational status were found to be significantly associated with knowledge about the COVID-19 vaccine at a *p* < 0.05. Study participants whose ages ≥49 years old were 1.6 times more likely to have good knowledge about the COVID-19 vaccine compared with the age group of 18–34 years (AOR = 1.643; 95% CI = 1.008–3.060). Study participants who had a college and above level of education were three times more likely to have good knowledge about the COVID-19 vaccine compared with those primary level of education (AOR = 3.002; 95% CI = 1.897–5.021) (Table 3).

**Table 3:** Factors associated with knowledge about COVID-19 vaccine among patients with chronic diseases, southern Ethiopia, 2021 (*N* = 409).

**Variables**	**Knowledge**		**COR (95% CI)**	**AOR (95% CI)**	***P*-value**
	**Good knowledge**	**Poor knowledge**			
**Sex**					
Male	206 (81.4%)	47 (18.6%)	1.425 (1.043–3.132)	2.001 (0.808–4.005)	0.402
Female	118 (75.6%)	38 (24.4%)	1	1	
**Residence**					
Urban	275 (82.3%)	59 (17.7%)	2.473 (1.432–4.879)	3.627 (0.750–5.440)	0.330
Rural	49 (65.3%)	26 (34.7%)	1	**1**	
**Age**					
18–34	31 (75.5%)	9 (22.5%)	1	**1**	
34–49	(15,185.8%)	25 (14.2%)	1.753 (0.543–1.781)	2.87 (0.889–4.120)	0.520
≥49	142 (73.6%)	51 (26.4%)	0.808 (0.401–2.320)	1.643 (1.008–3.060)^*^	**0.004**
**Educational status**					
Primary education (grade 1–8)	96 (82.1%)	21 (17.9%)	1	1	
Secondary education (grade 9–12)	122 (70.1%)	52 (29.9%)	0.513 (0.106–1.110)	1.106 (0.743–2.910)	0.380
College and above	106 (89.8%)	12 (10.2%)	1.932 (1.182–3.880)	3.002 (1.897–5.021)^*^	**0.000**

* Statistically significant at p < 0.05. The meaning of bold value was to show they were statistically significant category of the variable. In short to easily catchup the predictor variables.

### Attitude toward COVID-19 vaccine among patients with chronic diseases

Overall, two hundred ninety (70.9%) participants had a favorable attitude toward COVID-19 vaccines whereas 119 (29.1%) of them had an unfavorable attitude toward the COVID-19 vaccine among patients with chronic diseases.

### Intention to take COVID 19 vaccine among patients with chronic diseases

Two hundred thirty-eight (58.2%) of participants intended to take the COVID-19 vaccine. However, 171 (41.8%) of respondents were not intended to take the COVID-19 vaccine.

### Factors associated to take the COVID-19 vaccine among patients with chronic diseases

Educational status and knowledge were found to be significantly associated with intention to take the COVID-19 vaccine at *p* < 0.05. Study participants who had a college and above level of education were 1.5 times more likely to intend to take the COVID-19 vaccine compared with those who had a primary level of education (AOR = 1.510; 95% CI = 1.002–3.975). Study participants who had good knowledge about the COVID-19 vaccine were 3.6 times more likely to intend to take the COVID-19 vaccine compared with their counterparts (AOR = 3.560; 95% CI = 1.481–6.120) (Table 4).

**Table 4:** Factors associated with intention to take COVID-19 vaccine among patients with chronic diseases, southern Ethiopia, 2021 (*N* = 409).

**Variables**	**Intended to take the COVID-19 vaccine**		**COR (95% CI)**	**AOR (95% CI)**	***P*-value**
	**Yes**	**No**			
**Age**					
18–34	18 (45%)	22 (55%)	1	1	
34–49	116 (65.9%)	60 (34.1%)	2.363 (1.089–4.521)	2.516 (0.944–4.877)	0.630
≥ 49	104 (53.9%)	89 (46.1%)	1.428 (0.870–2.867)	2.001 (0.693–3.302)	0.420
**Educational status**					
Primary education	79 (65.7%)	38 (32.5%)	1	1	
Secondary education	113 (64.9%)	61 (35.1%)	0.891 (0.241–2.003)	1.061 (0.796–2.228)	0.316
College and above	46 (39%)	72 (61%)	0.307 (0.1008–1.841)	1.510 (1.002–3.975)^*^	**0.001**
**Monthly income in USD**					
<$40	111 (72.5%)	42 (27.5%)	1	1	
$40–$80	41 (48.8%)	43 (51.2%)	0.361 (0.064–0.830)	0.419 (0.207–1.954)	0.120
≥$80	86 (50%)	86 (50%)	0.378 (0.049–0.920)	0.651 (0.104–1.480)	0.09
**Knowledge about the vaccine**					
Good	192 (59.3%)	132 (40.7%)	1.233 (1.009–3.041)	3.560 (1.481–6.120)^*^	**0.000**
Poor	46 (54.1%)	39 (45.9%)	1	1	

* Statistically significant at p < 0.05. The meaning of bold value was to show they were statistically significant category of the variable. In short to easily catchup the predictor variables.

## Discussions

The COVID-19 pandemic has been a health issue of great concern since its start in 2020 and causes high morbidity and mortality, especially in the higher-risk population groups like the one in this study, patients with chronic diseases (32). Vaccination is not the only, but the best solution to controlling infectious diseases (33). However, while most people vaccinate according to the recommended schedule, success is being challenged by multiple factors, especially in Africa. some of the major challenges threatening the success of the COVID-19 vaccination rollout in most African countries include the slow onset of the vaccination exercise, limited funds, concerns around vaccine safety and uncertainties, storage requirements and regulatory hurdles for vaccines, limited shelf life of COVID-19 vaccines, inability to access vulnerable communities in a timely fashion, problems around the use of different vaccines, and wars and conflicts (34).

In this study, the overall magnitude of knowledge, attitude, and intention to take the COVID-19 vaccine and associated factors among patients with chronic diseases in southern Ethiopia were investigated. Age and educational status were predictor variables for knowledge about the COVID-19 vaccine. Educational status and knowledge were predictor variables for intention to take the COVID-19 vaccine.

In this study, 79.2% (95% CI = 69.3–84.3%) of the participants had good knowledge about COVID 19 vaccine. This study's findings were similar to that of a study conducted in Ethiopia's Gurage Zone (74%) (13). The finding was higher than the studies conducted in West India (35.5%), Illibabur and Bedelle Zones of Ethiopia (64%), Bangladesh (62.1%), Southwest Ethiopia (60.8%), Jordan (50%), and Malaysia (38%) (10, 11, 15, 16, 35, 36). The disparities may be due to differences in the study population. The study population of this study was people who have a chronic medical illness. These individuals have less immunity and they are at risk of contracting communicable diseases including COVID-19 infection. They become more concerned about their health so they may search for different information outlets. The majority (85.3%) of our respondents who ever heard about the COVID-19 vaccine support this. The other reason may be due to differences in educational status. All of our study participants had a primary and above level of education and 71.4% of them had a secondary and above level of education. It may be the case that people that are more educated are more knowledgeable and concerned about their health and wellbeing, through access to more information sources, and become more engaged in life events that could affect them (37).

However, it was lower than studies conducted in Greece (88.28%), Oman (88.4%), and Romania (95.15%) (12, 14, 17). The difference may be due to variations in study participants and socioeconomic status. In a study done in Greece, the study participants were health care workers. Health care workers are more knowledgeable than other professions (16). The above studies were done in economically better counties with a well-integrated health system than our setup where study participants had family physicians which are unthinkable to our setup. When comparing the age groups of 18–34 years old and 49 years old and above, study participants who were 49 years old and above were 1.6 times more likely to have good knowledge about the COVID-19 vaccine. The findings matched with research from India, Jordan, and Southwest Ethiopia (11, 16, 31, 36). This could be because older people are more likely than younger people to experience serious side effects; as a result, they get concerned and look for additional information about the vaccine.

Study participants who had college and above level of education were three times more likely to have good knowledge about the COVID-19 vaccine compared with those with primary levels of education. This was consistent with studies done in Malaysia, Jordan, Bangladesh, and Southwest Ethiopia (15, 16, 35, 36). It's possible that people with more education are more knowledgeable and worried about their health and wellbeing, and become more active in life events that may affect them (37), such as COVID-19 immunizations because they have access to more information sources.

The overall proportion of favorable attitudes of participants toward the COVID-19 vaccine was 70.9%. This finding was consistent with studies done in Belgium and Italy (17, 27). However, it was higher than studies in Ethiopia (44.7% and 24.2%), UK (36.9%), and Egypt (34.3%) (13, 28, 38, 39). The disparity may be due to differences in features of study participants. Although the proportion of the population that needs to be vaccinated to achieve herd immunity remains largely unknown, some have estimated that the herd immunity threshold for COVID-19 ranges from 60 to 70% (34, 40).

According to the finding of this study, 58.2% (95% CI = 51.0–61.2%) of study participants had intention to take COVID 19 vaccine. This finding was consistent with studies done in Dessie (59.4%), Gondar (54.8%), and the Kingdom of Saudi Arabia (52%) (18, 19, 25). It was lower than the findings of research conducted in Saudi Arabia (64.7%) and Ethiopia (64.9%) (20, 21). The disparities may be because of a difference in study design that is institutionally based in this study whereas web-based national survey in the previous studies. However, it was higher than studies conducted in Butajira (33.7%), Woliata Sodo town (46.1%), and national level Ethiopia (31.4%) (22–24). The difference could be due to the difference in the study population. In this study, the study population was patients with chronic diseases whereas not for the above studies. Patients with chronic diseases have a high vaccine acceptance rate (41–43).

Study participants who had college and above level of education were 1.5 times more likely to intend to take the COVID-19 vaccine compared with those who had a primary level of education. This finding was supported by studies done in Ethiopia at a national level, Gondar, and the Kingdom of Saudi Arabia (18, 21, 22, 25). This is because education raises public awareness of the vaccine's health benefits. Several studies have found that more years of training are linked to greater acceptance of the COVID-19 vaccination (44, 45).

Study participants who had good knowledge about the COVID-19 vaccine were 3.6 times more likely to intend to take the COVID-19 vaccine compared with their counterparts. This was in line with studies done in Dessie, Butajira, and Ethiopia at the national level (19, 21, 22). This might be because knowing is a prerequisite to developing intention. If participants are aware of the advantages of vaccines through different outlets, this facilitates informed decisions and could be intended to take the vaccine. The effectiveness of vaccines relies on both clinical efficacy and a community's knowledge. During vaccine promotion, lack of community support due to poor knowledge and perceptions resulted in poor community uptake while others reject vaccines (46).

### Limitation of study

This study has some limitations. The cross-sectional design of the study inherently limits determining the direction of causation, between dependent and independent variables. Besides, since it was a quantitative approach, it does not explore the participants' deep feelings, acceptability and reason for refusal, or beliefs about the COVID-19 vaccine, unlike a qualitative approach. In addition, there might be a potential for social desirability bias, which means participants may have been inclined to provide socially desirable responses rather than describe actual beliefs and practices.

## Conclusions and recommendations

Knowledge and attitudes toward the COVID-19 vaccine among patients with chronic disease were high relative to other studies. However, the intention to take the COVID-19 vaccine was low to achieve herd immunity.

Age and educational status were predictor variables for knowledge about the COVID-19 vaccine. Educational status and knowledge were predictor variables for intention to take the vaccine among patients with chronic diseases.

Holistic and multi-sectoral partnership is necessary for a successful COVID-19 vaccination campaign. Further health education and communication are very crucial methods to improve vaccine acceptance and lastly to achieve herd immunity.

## Data availability statement

The raw data supporting the conclusions of this article will be made available by the authors, without undue reservation.

## Ethics statement

The studies involving human participants were reviewed and approved by Wolaita Sodo University Institutional Review Board (IRB). The patients/participants provided their written informed consent to participate in this study.

## Author contributions

All authors made considerable contributions to conception and design, acquisition of data, or evaluation and interpretation of data, took section in drafting the article or revising it significantly for necessary intellectual content, agreed to put up to the current journal, gave ultimate approval of the version to be published, and agree to be responsible for all elements of the work.

## Conflict of interest

The authors declare that the research was conducted in the absence of any commercial or financial relationships that could be construed as a potential conflict of interest.

## Publisher's note

All claims expressed in this article are solely those of the authors and do not necessarily represent those of their affiliated organizations, or those of the publisher, the editors and the reviewers. Any product that may be evaluated in this article, or claim that may be made by its manufacturer, is not guaranteed or endorsed by the publisher.

## Abbreviations

ARDS: acute respiratory distress syndrome
AOR: adjusted odds ratio
CI: confidence interval
COVID-19: corona virus disease−19
ICU: intensive care unit
SD: standard deviation
VIF: variance inflation factor
WSUTRH: woliata sodo university teaching and referral hospital.
